# Prevalence of depression and associated factors among hemodialyzed patients in Jazan area, Saudi Arabia: a cross-sectional study

**DOI:** 10.1108/MIJ-02-2020-0004

**Published:** 2020-05-20

**Authors:** Asim Othayq, Abdulwahab Aqeeli

**Affiliations:** Department of Health Affairs, Saudi Arabia Ministry of Health, Riyadh, Saudi Arabia; Department of Family and Community Medicine, Jazan University, Jazan, Saudi Arabia

**Keywords:** Saudi Arabia, Depression, Hemodialyzed patients, DASS-42, kidney disease, hemodialysis

## Abstract

**Purpose:**

This study aims to evaluate the prevalence of depression and associated risk factors among patients on hemodialysis in Jazan area, Saudi Arabia.

**Design/methodology/approach:**

The study was conducted on 211 randomly selected hemodialysis patients in Jazan area, Saudi Arabia, using an observational cross-sectional design. Patients were screened for depressive symptoms using the depression, anxiety and stress scale 42 (DASS-42). Descriptive statistics were used to present sociodemographic data. Multiple logistic regression was implemented to identify the predictors of depression. Data were entered and analyzed using SPSS 22.0 software.

**Findings:**

The study found the overall prevalence of depression among patients on hemodialysis to be 43.6 per cent. Of them, 12.8 per cent were mildly depressed, 15.6 per cent were moderately depressed and 15.1 per cent fell in the severe or extremely severe category. Depression was significantly associated with marital status, education level and the presence of sleep disturbances. The study indicates a high prevalence of depressive symptoms among patients on hemodialysis in Jazan. A higher rate of depressive symptoms was observed in currently unmarried, lower-educated patients and those with sleep disturbance.

**Originality/value:**

Periodic evaluation of patients on hemodialysis for depression is needed to allow for early intervention.

## Introduction

End-stage renal disease (ESRD) is a progressive and irreversible loss of kidney function over a long period and sufficiently severe to require maintenance dialysis or kidney transplantation to maintain health or life (Müller *et al.*, 2010). Like numerous chronic diseases, ESRD may influence the psychological status of the patients (Feroze *et al.*, 2010). Psychiatric illnesses, including depression in patients with ESRD, have its effect on the morbidity and healthcare costs among patients with ESRD. The term “psychonephrology” has been introduced by Levy to refer to psychiatric problems of patients suffering from chronic kidney disease and particularly those with kidney failure on maintenance dialysis or who underwent transplantation (Levy, 2008).

Depression is considered as the most common psychological illness among hemodialysis patients. Among the general population, the prevalence of major depression ranged between 1.1 and 23 per cent (Weissman *et al.*, 1996). However, among ESRD patients, it ranged between 20 and 30 per cent (Chilcot *et al.*, 2008) and may reach up to 60.5 per cent (Kao *et al.*, 2009). In Saudi Arabia, the prevalence of depression among ESRD patients on hemodialysis ranged between 23.3 and 83.5 per cent (Turkistani *et al.*, 2014; Saeed *et al.*, 2012; AlDukhayel, 2015; Joshwa *et al.*, 2012).

Despite the high incidence of depression among hemodialysis patients, its diagnosis is often overlooked (Cukor *et al.*, 2007). Diagnosis of depression in these patients is essential as depression in chronic diseases, in general, has been associated with lack of adherence to the therapy, high suicidal tendencies and poor survival rates (Katon *et al.*, 2007; Diefenthaeler *et al.*, 2008; Hedayati *et al.*, 2005; Kimmel *et al.*, 1993). Moreover, a strong association has been observed between depression and longitudinal outcomes among hemodialysis patients, including poor adherence to treatment, impaired quality of life and higher mortality rates (Drayer *et al.*, 2006; Li *et al.*, 2016). In addition, depression among hemodialysis patients is associated with higher rates of hospital admission and a greater likelihood of emergency department (ER) visits (Tavallaii *et al.*, 2009; Hedayati *et al.*, 2008).

Few studies that have identified the prevalence of depression, along with their associated factors, among patients on hemodialysis in the Kingdom of Saudi Arabia. However, there is limited information regarding the prevalence of depression and its associated risk factors among patients on hemodialysis in the Southern area in Saudi Arabia. Therefore, this study aimed to estimate the prevalence of depression and its associated risk factors among patients on hemodialysis in Jazan area, Saudi Arabia.

## Material and methods

### Study area, design and population

A cross-sectional study was conducted among patients on hemodialysis in Jazan province, one of the 13 provinces of Saudi Arabia. It is located in the southwestern border of the country. It has a total population of approximately 1.5 million according to the 2015 census. Jazan has eight governmental hemodialysis centers serving 842 registered patients at the time of the study.

### Sampling method and sample size

A sample of 229 out of 842 registered hemodialysis patients was recruited by systematic random sampling. The sample size was calculated based on the following parameters; *P* = 23.3 per cent (the lowest reported depression prevalence among hemodialysis patients in Saudi Arabia), 95 per cent confidence level, a margin of error below 5 per cent and nonresponse rate of 20 per cent. Patients were recruited from three randomly selected out of eight governmental hemodialysis centers in Jazan.

### Data collection and study instrument

Data were collected by trained nurses through face-to-face interview using an Arabic questionnaire while patients were waiting for their dialysis session. The questionnaire was composed of two main parts; the first part contained patient’s demographics (age, gender, marital status, living status, education, employment status), comorbid history (diabetes and hypertension), history of sleep disturbance, current smoking status, physical activity status and duration of dialysis. The second part contained depression questions adapted from the Arabic version of the depression, anxiety and stress scale 42 (DASS-42). The DASS-42 consists of a 42-item self-report inventory designed to screen for the presence and severity of symptoms of depression, anxiety and stress among people as young as 18 (Lovibond and Lovibond, 1995). This scale was psychometrically validated to the Arabic culture (Moussa *et al.*, 2017). This screening and outcome measure reflects the experience of the person over the previous seven days. Depression scale contained 14 questions presented as a four-point Likert scale, with 0 being does not apply and 3 being very applicable/applies most of the time. The scores were calculated by summing the scores of the depression questions.

### Data analysis and management

Data were collected and verified by hand before data entry. The statistical Package for Social Sciences (SPSS) software version 22.0 (SPSS Inc., Chicago, IL, USA) was used for data entry and analysis. Descriptive statistics and inferential statistics were applied. Sociodemographic factors were presented by frequency and percentage, except for age and the duration of hemodialysis, which were presented by mean and SD. Univariate analysis to determine the association of each variable with depression was also implemented. Finally, multivariate logistic regression analysis was performed to identify predictors of depression among patients after controlling for confounders. The results were expressed as adjusted odds ratio and 95 per cent confidence intervals. All statistical tests were two-sided, and a *p*-value less than 0.05 was considered statistically significant.

### Ethical consideration

This study was conducted in accordance with the ethical principles within the Kingdom of Saudi Arabia. Ethical approval was obtained from the Faculty of Medicine Ethical Committee at Jazan University. The privacy and confidentiality of the data were maintained throughout the study period. Patients read, understood and signed informed consent.

## Results

A total of 211 hemodialysis patients participated in the study (response rate = 92.1 per cent). Their sociodemographic and health-related characteristics are summarized in Table 1. Patients’ age ranged between 16 and 90 years (mean ± SD: 48.5 ± 14.8). Almost half of them (47 per cent) were in the age group 31-50 years, whereas 17.5 per cent of them were aged over 60 years. Female patients represent 59.7 per cent, with almost two-thirds of them (66.4 per cent) being married. Majority of patients live with others (95.7 per cent) and were not working (85.3 per cent). Regarding educational level, 60.7 per cent of the participants were illiterate, whereas 9.4 per cent were at least university graduates. The prevalence of current smoking, diabetes and hypertension among patients were 4.7, 23.2 and 68.2 per cent, respectively. Moreover, 55.9 per cent of the patients had sleep disturbances, and 31.3 per cent were physically active. The duration of hemodialysis ranged between one month and 31 years with a mean of five years and a standard deviation of 5.1. Approximately, 27.5 per cent of the patients had hemodialysis duration that exceeded five years, whereas 20.9 per cent of them had a duration of one year or less of hemodialysis. Depression was more likely to be reported among patients who were female (50.8 per cent, *p* = 0.010), divorced or widowed (72.4 per cent, *p* = 0.001), illiterate (53.1 per cent, *p* = 0.002), had sleep disturbance (57.6 per cent, p < 0.001) and were physically inactive (49.7 per cent, *p* = 0.009). Smoking history, histories of diabetes and hypertension and duration of hemodialysis were not significantly associated with depression.

The mean score of depression symptoms was at normal level (Table 2). The symptoms of depression were reported among 43.6 per cent of hemodialysis patients. The prevalence of mild, moderate, severe and extremely severe symptoms of depression were 12.8, 15.6, 12.3 and 2.8 per cent, respectively.

Table 3 presents the results of the multivariate logistic regression analysis. The analysis revealed that single (OR = 3.04, 95 per cent CI: 1.32-6.97, *p* = 0.009) and divorced/widowed marital status (OR = 3.35, 95 per cent CI: 1.29-8.68, *p* = 0.013) strongly predicted the risk of depression. Other predictors of risk for depression included secondary school education, which significantly lowered risk for depression (OR = 0.36, 95 per cent CI: 0.16-0.80, *p* = 0.012) and sleeping disturbances, which was the strongest predictor for the risk of depression. (OR = 3.75, 95 per cent CI: 2.01-7.27, *p* = 0.000).

## Discussion

The aim of this study was to identify the prevalence of depression and related risk factors among patients on hemodialysis in Jazan region, Saudi Arabia. Our study revealed that depression, regardless of its severity, was present among 43.6 per cent of the patients on hemodialysis. This estimate is higher than that reported by Turkistani *et al.* in their study in Makkah (23.3 per cent) using the Hospital Anxiety and Depression Scale (HADS) (Turkistani *et al.*, 2014). On the other hand, it was lower than that observed by Saeed and his colleagues on patients with ESRD on hemodialysis using the Beck’s Depression Inventory (BDI-II) scoring (75 per cent) (Saeed *et al.*, 2012). Globally, the prevalence rate of depression among patients on hemodialysis ranged between 20 and 60.5 per cent (Chilcot *et al.*, 2008; Kao *et al.*, 2009) This difference in the prevalence of depression could be attributed to different tools used for estimating the prevalence of depression as well as sample characteristics.

In studying the sociodemographic factors, marital status, education level and sleep disturbances were associated with depression. Our findings showed that being unmarried was highly correlated with increased depression prevalence rate. This is the opposite to what Saeed *et al.* reported in their study where married patients were more likely to have depression (Saeed *et al.*, 2012). This difference could be attributed to the fact that married individuals hold more responsibility to their families. On the other hand, gender and physical activity were not correlated with depression contradicting previous studies (Li *et al.*, 2016; Fischer *et al.*, 2010; Chiang *et al.*, 2013; Bayat *et al.*, 2011).

There is lack of evidence on the relationship between sleep disturbances and depression among ESRD patients. However, a recently online published article has found that insomnia is related to depression among hemodialysis patients in Indonesia (Lufiyani *et al.*, 2019). Furthermore, insomnia and sleep complaints were common among ESRD patients on hemodialysis (Walker *et al.*, 1995; Hanly, 2004; Parker, 2003). Additionally, a study of the general population suggested a causative link between insomnia and depression (Gillin, 1998). Moreover, a meta-analysis by Chiara *et al.* found a twofold increase in the risk of depression in healthy individuals (Baglioni *et al.*, 2011). In our study, the presence of sleep disturbance was significantly associated with depression. Patients who had sleeping disturbance were at almost a fourfold increased risk for depression compared to those without sleep disturbances. Consequently, it is clinically significant for patients on hemodialysis to be screened for sleep problems to protect them from depression.

From the estimates mentioned above, it is clear that depression is increasingly prevalent among patients on hemodialysis. Hence, the implications of these results are very important for early intervention to limit the impact of depression and improve the quality of life for those patients. However, the recognition and management of depression are still suboptimal in routine care of those patients (Hedayati *et al.*, 2005). Therefore, healthcare providers must be aware of the existence of depression among patients on hemodialysis and be able to provide standard care procedures to them and implement early screening to identify and refer high-risk cases (Fischer *et al.*, 2010).

## Limitations

The authors acknowledge the following limitations of the present study. First, this study used a cross-sectional design; hence, we cannot infer causality from our findings. Second, the sleep disturbance and physical activity were examined with only a single-item question rather than using structured scale, which may have been more robust. Finally, the DASS-42 scale is used to screen for depressive symptoms rather than make a diagnosis of depression; therefore, the current findings should be interpreted with caution.

## Conclusions

Depression was highly prevalent among patients on hemodialysis in Jazan region, Saudi Arabia. Current unmarried, lower-educated patients as well as those having sleep disorders were more likely to have depression. Our findings draw attention to the importance of screening hemodialyzed patients for depression to intervene early.

## Figures and Tables

**Table I:** Sociodemographic and health-related characteristics of patients on hemodialysis in Jazan area

**Characteristics**	*n* (%)	Depression *n* (%)	Test value (*χ^2^*)	*df*	*p*-value
***Gender***					
**Male**	85 (40.3)	28 (32.9)	6.58	1	0.010
**Female**	126 (59.7)	64 (50.8)			
***Marital status***					
**Married**	140 (66.4)	50 (35.7)	14.03	2	0.001
**Single** **Divorced/widowed**	42 (19.9) 29 (13.7)	21 (50.0) 21 (72.4)			
***Working status***					
**Working**	31 (14.7)	10 (32.3)	1.90	1	0.168
**Not working**	180 (85.3)	82 (45.6)			
***Educational level***					
**Illiterate**	128 (60.7)	68 (53.1)	12.40	2	0.002
**Secondary school or below**	63 (29.9)	17 (27.0)			
**University or above**	20 (9.4)	7 (35.0)			
***Cigarette smoking***					
**Smoker**	9 (4.3)	2 (20.0)	2.86	2	0.239
**Ex-smoker**	26 (12.3)	10 (38.5)			
**Non-smoker**	176 (83.4)	80 (45.7)			
***Diabetes***					
**Yes**	49 (23.2)	21 (42.9)	0.01	1	0.904
**No**	162 (76.8)	71 (43.8)			
***Hypertension***					
**Yes**	144 (68.2)	62 (43.1)	0.06	1	0.815
**No**	67 (31.8)	30 (44.8)			
***Sleep disturbance***					
**Yes**	118 (55.9)	68 (57.6)	21.42	1	<0.001
**No**	93 (44.1)	24 (25.8)			
***Physical activity***					
**Yes**	66 (31.3)	20 (30.3)	6.91	1	0.009
**No**	145 (58.7)	72 (49.7)			

**Table II:** Prevalence and score severity ratings of depressive symptoms among patients on hemodialysis in Jazan area

**Category**	Score severity ratings	Frequency (%)
**Normal**	0-9	119 (56.4)
**Mild**	10-13	27 (12.8)
**Moderate**	14-20	33 (15.6)
**Severe**	21-27	26 (12.3)
**Extremely severe**	28+	6 (2.8)
**Mean ± SD**	9.6 ± 8.5	

**Table III:** Multivariate logistic regression analysis of risk factors for depression among patients on hemodialysis in Jazan area

**Characteristics**	Adjusted odds ratio	95% confidence interval		*p*-value
		**Upper**	Lower	
***Gender***				
**Male (*n* = 85)**	1			
**Female (*n* = 126)**	0.95	0.43	2.11	0.894
***Marital status***				
**Married (*n* = 140)**	1			
**Single (*n* = 42)**	3.04	1.32	6.97	0.009
**Divorced/widowed (*n* = 29)**	3.35	1.29	8.68	0.013
***Educational level***				
**Illiterate (*n* = 128)**	1			
**Secondary school or below (*n* = 63)**	0.36	0.16	0.80	0.012
**University or above (*n* = 20)**	0.67	0.23	1.96	0.461
***Smoking history***				
**Smoker (*n* = 10)**	0.57	0.09	3.56	0.547
**Ex-smoker (*n* = 26)**	1.30	0.43	3.93	0.643
**Non-smoker (*n* = 175)**	1			
***Diabetes mellitus***				
**Yes (*n* = 49)**	0.93	0.43	1.99	0.843
**No (*n* = 162)**	1			
***Hypertension***				
**Yes (*n* = 144)**	0.89	0.44	1.79	0.745
**No (*n* = 67)**	1			
***Sleep disturbance***				
**Yes (*n* = 118)**	3.75	1.90	7.37	0.000
**No (*n* = 93)**	1			
***Physical activity***				
**Yes (*n* = 66)**	0.79	0.39	1.63	0.530
**No (*n* = 145)**	1			
